# A novel method to quantify arterial pulse waveform morphology: attractor reconstruction for physiologists and clinicians

**DOI:** 10.1088/1361-6579/aae46a

**Published:** 2018-10-30

**Authors:** Manasi Nandi, Jenny Venton, Philip J Aston

**Affiliations:** 1 School of Cancer and Pharmaceutical Sciences, Faculty of Life Sciences and Medicine, King’s College London, Franklin Wilkins Building, 150 Stamford Street, London SE1 9NH, United Kingdom; 2 School of Cardiovascular Medicine and Sciences, Faculty of Life Sciences and Medicine, King’s College London, Franklin Wilkins Building, 150 Stamford Street, London SE1 9NH, United Kingdom; 3 Department of Mathematics, University of Surrey, Guildford, Surrey GU2 7XH, United Kingdom; manasi.nandi@kcl.ac.uk

**Keywords:** attractor reconstruction, cardiovascular physiology, morphology, variability, arterial pulse waveform

## Abstract

Current arterial pulse monitoring systems capture data at high frequencies (100–1000 Hz). However, they typically report averaged or low frequency summary data such as heart rate and systolic, mean and diastolic blood pressure. In doing so, a potential wealth of information contained in the high-fidelity waveform data is discarded, data which has long been known to contain useful information on cardiovascular performance.

Here we summarise a new mathematical method, attractor reconstruction, which enables the quantification of arterial waveform shape and variability in real-time. The method can handle long streams of non-stationary data and does not require preprocessing of the raw physiological data by the end user. Whilst the detailed mathematical proofs have been described elsewhere (Aston *et al* 2008 *Physiol. Meas*. **39**), the authors were motivated to write a summary of the method and its potential utility for biomedical researchers, physiologists and clinician readers.

Here we illustrate how this new method may supplement and potentially enhance the sensitivity of detecting cardiovascular disturbances, to aid with biomedical research and clinical decision making.

## 1. Introduction

Arterial pulse waveform analysis has a long tradition dating back to the mid-19th century with key figures including Etienne Jules Marey, an intern at Hôpital Cochin, Paris and Frederick Mohamed, a physician at Guy’s Hospital, London (O’Rourke 1992, Bartels *et al* 2016).

**Figure 1.**
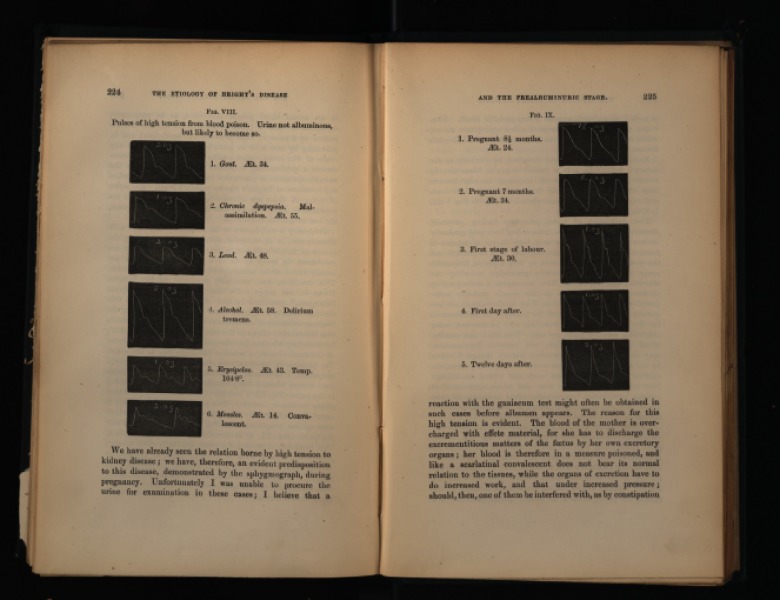
Images captured by a sphygmograph from patients with different pathologies or during pregnancy illustrating waveform morphology changes (Mahomed 1874). Images courtesy of King’s College London, Foyle Special Collections Library.

Marey adapted a piece of apparatus, initially developed by Karl Vierordt of Tubingen, which enabled the shape of an arterial pulse waveform to be captured and analysed using a non-invasive device placed on the forearm (Lawrence 1979, Ferro *et al* 2012). This ‘sphygmograph’ was later put into clinical use by Frederick Mohamed, who published a series of elegant studies detailing how differences in pulse waveform morphology were apparent between radial and carotid sampling positions, essential hypertension and chronic nephritis, and further described changes in waveform morphology pre and post-partum, during fever, ageing and following infection, amongst others (Mahomed 1872, 1874, O’Rourke 1992) (figure 1).

Since that time, scientists and clinicians have studied, in more detail, elements of the arterial pulse waveform and behaviour beyond routine measurements of maximum, minimum and mean arterial pressures and heart rate.

Numerous waveform morphology, variability and dynamic features have been described, including pulse pressure, upstroke gradients, augmentation pressure, dichrotic notch positioning, arterial ‘swings’ and pulse wave analysis and velocity (Laurent *et al* 2006, Nichols *et al* 2008, Weber *et al* 2010, Ben-Shlomo *et al* 2014, Nirmalan and Dark 2014, Segers *et al* 2017). Quantification of these features enhances the understanding of cardiovascular physiology and event risk and provides information about cardiovascular state, e.g. cardiac dysfunction, fluid loading and vascular resistance.

Efforts have been made to reach consensus on how the arterial pulse waveform should be analysed and the physiological significance of individual morphological characteristics (Nirmalan and Dark 2014, Townsend *et al* 2015, Hametner and Wassertheurer 2017, Segers *et al* 2017). It is recognised that factors such as height, sex, heart rate, age and exercise, in addition to disease, can greatly influence the morphology of the pulse wave (Smulyan *et al* 1998, Wilkinson *et al* 2000, McEniery *et al* 2008, Nichols *et al* 2011) as do certain pharmacological agents (McVeigh *et al* 2001, Townsend *et al* 2015).

Detailed pulse waveform morphology analysis thus represents a valuable supplement to routine cardiovascular measurements to aid biomedical research, clinical decision making and patient management. However, these consensus papers suggest that a degree of qualitative assessment of the waveform shape is required by a trained individual.

This type of interpretation may be impracticable in many laboratory or clinical settings, where a specialist may not always be available or, indeed, the feature changes are not always obvious to the naked eye. Secondly, these techniques often focus on a snapshot window of data rather than looking at longer data streams (e.g. a patient trajectory over hours or days) which may mean important waveform feature changes are missed. Thirdly, there is great inter-individual variation even amongst healthy individuals, which makes setting any broad guideline measures relating to pathological changes in pulse wave morphology, challenging. Thus, whilst there are many technological advances in waveform capture and algorithms that derive potentially useful measures, translating these into readily usable and understandable formats is by no means straightforward.

## 2. Current arterial pulse waveform analysis

Whilst there are methods available for analysing arterial pulse waveforms, including morphology analysis, many require a degree of preprocessing, some require assumptions to be made and the full range of data is not always being exploited (Segers *et al* 2017). Below we have identified what we believe to be some of the issues that need to be addressed.

### 2.1. First issue: not using all of the high-fidelity data

High-frequency sampling of arterial blood pressure waveforms from monitoring devices generates too many numerical values for a clinician or researcher to meaningfully interpret in real time. A typical hospital monitor samples data at 125 Hz (125 data points per second) whilst preclinical devices, such as radiotelemetry implants, are often set to record at 500–1000 Hz. To facilitate meaningful interpretation of the sampled data, these devices provide regular averages of the maximum and minimum pressures, rate etc which are displayed to the end user. This provides the blood pressure trajectory over a given time period, which in turn facilitates clinical decision making in patients or interpretation of experimental interventions (e.g. novel pharmacotherapies) in preclinical research.

This process of averaging is a very simplified analysis of the data.

Let us take the example of a patient with a cannula in the radial artery coupled to a bedside real time blood pressure monitor set to 125 Hz. If the patient is monitored over a 10 s period (e.g. 10 heart beats/pulse waves) the monitor will have recorded 1250 data points.

Displaying 1250 numbers across the screen over a 10 s period would be impossible for a clinical staff member to read or interpret meaningfully. Therefore the device averages the 10 s data stream (10 pulse waves) and might display the following *averaged* numerical values.

1. Average maximum peak value of waves (systolic pressure).
2. Average minimum trough value of waves (diastolic pressure).
3. Average mean arterial pressure.
4. Average height of waves (pulse pressure).
5. Number of peaks (heart rate).

However, the above averaged measures ignore the majority of sampled data points, focussing on the top and bottom of the waveform only. They therefore provide no information pertaining to the intermediate points of the waveform which correspond to the shape of the wave.

### 2.2. Second issue: plotting the waveform data against time

**Figure 2.**
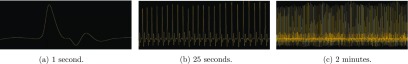
The images show an arterial pulse waveform data stream obtained by a non-invasive finger tip monitor (Finapres) over (a) 1 s, (b)  ∼25 s and (c)  ∼2 min. The detailed morphology of the waveform is not visible when viewed over longer periods of time.

We typically view arterial waveform data by plotting it against a time axis (time series data). By doing so, the ability to see and describe any changes in the shape and variability of the waveform becomes extremely difficult when looking over long periods of time (figure 2). Consequently, waveform shape can only be quantified by focussing on a short section of the data from a much longer data stream.

### 2.3. Third issue: baseline wander and noise

Baseline wander can occur in an arterial pulse wave due to physical and physiological interference caused by respiration and movement. This can corrupt the signal such that estimation of arterial and pulse pressures may be impacted. Further, this ‘noise’ impedes visualisation of waveform shape and variability. Other complications can arise, such as damping caused by catheter microbubbles or baseline drift arising from incorrect zeroing of the recording device. Both can lead to under or over estimates of mean arterial, systolic, diastolic or pulse pressures (Esper and Pinsky 2014). To address this issue, baseline wander is sometimes removed, especially on ECG signals (Fedotov and Akulova 2015, Li *et al* 2017, 2018). However, editing the original signal in this way may exclude important information from the data.

## 3. A new way of quantifying the arterial pulse waveform morphology

To overcome these three issues, we have developed a new way of visualising and quantifying physiological waveform data. Here we focus on the application of this method to continuous arterial blood pressure waveform data but it is important to note that this method can equally be applied to ECG, pulse oximetry, respiratory impedance and other waveforms that are approximately periodic (Charlton *et al* 2015, Aston *et al* 2018, Lyle *et al* 2018).

The method combines the disciplines of mathematics (nonlinear dynamical systems) with cardiovascular physiology. This method allows the quantification of numerous morphological features and the variability of physiological waveforms. We summarise and describe the key points of the method below, but a detailed explanation can be found in appendices A–E. A short video summary of the method can be accessed online and a glossary of key terms is available at the end of this paper^4^4http://ehealth.kcl.ac.uk/cardiomorph/..

**Figure 3.**
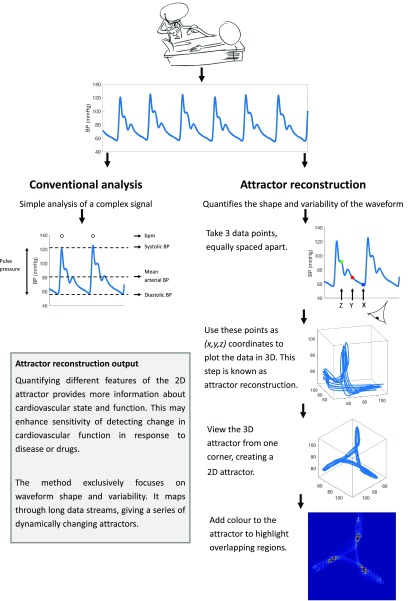
Stages of the attractor reconstruction method applied to an arterial pulse waveform. Human arterial pulse waveform data collected with a finger tip monitor (Finapres).

This new method replots and visualises the raw waveform data in a different way, allowing new information to be extracted from a routine signal (see figure 3). The method plots the raw data in three-dimensions using a technique known as ‘attractor reconstruction using delay coordinates’, which was published in a seminal paper by Dutch mathematician Floris Takens in 1981 (Takens 1981). Looking at this three-dimensional plot of the data from one corner converts it to a two-dimensional image. By doing so this two-dimensional image enables the unique quantification of arterial pulse waveform shape and variability over time.

The attractor reconstruction method addresses the issues raised in section 2 in the following way.

Issue 1: **This method uses every single data point on the entire sampled waveform.**
  This means the end user does not need to edit the raw data before attractor reconstruction. In this way, the method is resistant to bias introduction, as no preselection or post processing is required. The data is used in its entirety barring non-physiological artefacts. Of course, the quality of the data should always be examined to distinguish between artefactual noise (e.g. electrical or mechanical disturbances) versus physiological noise (e.g. respiratory changes, physiological, pharmacological perturbations).
Issue 2: **This method replots the data in three-dimensions, removing the time axis.**
  This means that all of the data, sampled over any time scale, is now constrained within the cube of fixed size. It is now possible to quantify how the waveform’s shape and variability changes over a long period of time.
Issue 3: **The method is unaffected by changes in baseline wander in the signal.**
  This method ignores movement in the *y*-axis of the raw signal. Baseline wander is therefore not an issue for the attractor reconstruction method. A more detailed explanation can be found below. Once again, the quality of the data should always be examined to exclude non-physiological artefacts.

In addition the attractor reconstruction method can also

**Be used on any approximately periodic signal and is not limited to arterial blood pressure.**
  Any approximately periodic (repeating) waveform such as arterial pressure, ECG, pulse oximetry, respiratory impedance or central venous pressure is suitable for attractor reconstruction providing it is sampled at an appropriate frequency (Aston *et al* 2014, Charlton *et al* 2015, Aston *et al* 2018, Lyle *et al* 2018).

### 3.1. Analogy to aid understanding

**Figure 4.**
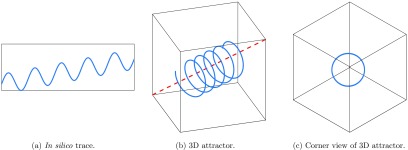
Relationship between (a) an *in silico* trace with baseline wander, (b) the corresponding three-dimensional attractor and (c) the same attractor rotated and viewed down one corner in the direction indicated by the red dotted line. Note that in (c) the red dotted line is pointing directly out of the page.

**Figure A4.**
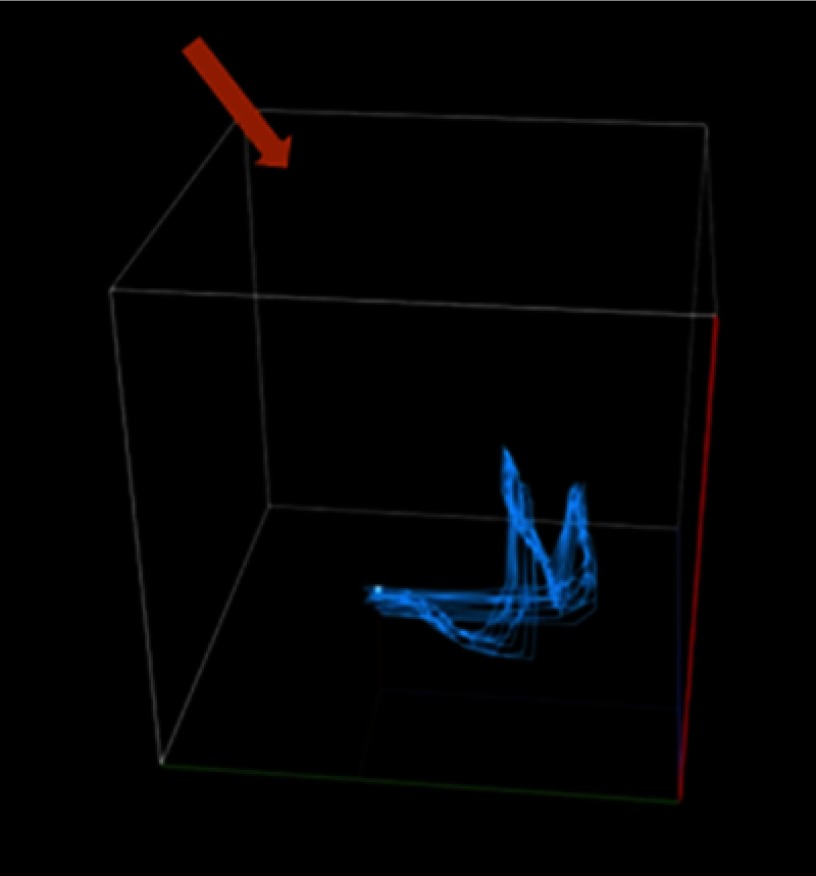
Numerous, overlapping loops in the cube create a three-dimensional shape, known as an attractor.

**Figure A5.**
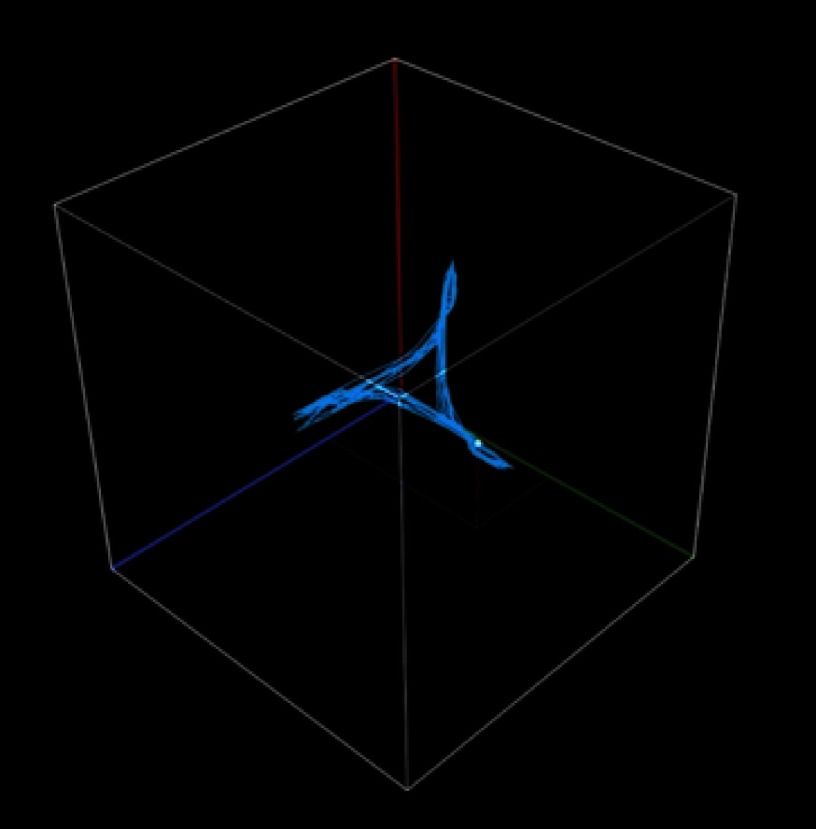
View of the three-dimensional attractor from one corner of the cube.

Attractor reconstruction generates a three-dimensional attractor using all of the high-fidelity waveform data. When viewed in 3D, it looks chaotic and difficult to quantify (see appendix figure A4). At this stage it encompasses all of the variation arising in terms of baseline wander and changes in cardiac/vascular function during consecutive cardiac cycles. By rotating the chaotic and noisy attractor and viewing down one particular corner (as indicated in appendix figure A4 and the red dotted line in figure 4(b)), it becomes a two-dimensional attractor (appendix figure A5). Now much of the chaos is no longer apparent and we are left with a structured triangular shape which can be quantified more easily. Importantly nothing has been removed or deleted, we are merely viewing the data from one direction which means the noisiest part is no longer obvious by eye.

An analogy would be if a multi-coloured slinky spring was stretched and mounted in a glass cube from one corner to the opposite corner. Viewing the glass cube from most angles would allow many of the features and colours of the stretched spring to be seen (figure 4(b)). However, looking directly down one corner of the cube, the spring would appear as a thick circle. This is illustrated in figure 4(c). It is important to note that this viewing direction is always the same; see Aston *et al* (2018) and appendix C for more detail.

We have shown that the noisiest part of the three-dimensional attractor arises from baseline wander, i.e. movement in the *y*-axis of the raw signal in figure 4(a). Therefore, counterintuitively, our method factors out the very thing we usually focus on—the changes in absolute maximum and minimum pressures.

However, by factoring out fluctuations in absolute pressure, attractor reconstruction focusses solely on the morphology of the arterial pulse waveform. In other words, it focusses on waveform contours that relate to cardiac contraction, wave reflections and alterations in resistance, and compliance of the vasculature. This is not to say that conventional measures of absolute pressure are not of value. Rather that attractor reconstruction allows us to extract additional new information from the same signal. We hypothesize this will provide more detailed information about cardiovascular performance and may enhance the sensitivity of detecting changes in experimental and clinical settings.

Each and every change on the arterial pulse wave that occurs in response to activity, aging, disease or drug intervention will, by definition, produce a corresponding change in specific features of the two-dimensional attractor. Importantly, whilst we are limited to using still images in this paper, in reality, using a moving window to track through the arterial pulse waveform data over time generates a dynamically changing two-dimensional attractor. Hence, both the absolute morphological features and their variability over time can be quantified as the system tracks through a long stream of patient or laboratory arterial pulse waveform data.

### 3.2. Take home message

Attractor reconstruction provides a new way of quantifying physiological waveform shape and variability. This method uses all of the raw waveform data and replots them in three-dimensions to generate an ‘attractor’.

Viewing the three-dimensional attractor from one corner gives a two-dimensional attractor. It is important to note that no data has been discarded; it is simply that the greatest variation appears to occur mainly in one direction when viewing the data in three-dimensions.

As the arterial pulse waveform shape changes over time, a new attractor is generated for each consecutive time window. This allows the attractor to track physiological changes through time.

Features of the two-dimensional attractor directly correlate with features of the arterial pulse waveform morphology and variability and we can now quantify these. Examples of how the attractor is quantified and further details of this are given in section 4.

## 4. What does the attractor tell us?

There are a very large number of ways to quantify the features of the two-dimensional attractor, including quantitative measurements of the width of the arms, the overall size, the highest density region, the degree of rotation—and so on. Through systematic studies using biological waveforms and simulated signals, we now have a better understanding of the physiological meaning of certain attractor features, as summarised in table 1. However, interpreting all the various attractor features in a particular clinical context would be a complicated process. Furthermore, as the attractor tracks through long streams of data, it will dynamically change—these changes will correlate to variations in the arterial pulse waveform over time.

**Table 1.** Examples of waveform features, corresponding attractor features and the physiological interpretation of this. Adapted from table 1, Aston *et al* (2018).

Blood pressure waveform feature	Attractor feature	Physiological interpretation
Decrease in cycle length	No change in attractor but average cycle length (or heart rate) traced against time	Increase in heart rate
Increase in amplitude	Attractor size increases	Increase in pulse pressure
Increased concavity of downstroke	Clockwise rotation of the attractor	Decreased resistance and compliance of peripheral vasculature
Increased convexity of upstroke	Non-uniform density along the edges	Increased force of cardiac contraction
Downstroke variability	Variability in right hand side of attractor	Variability in cardiac contraction
Waveform almost periodic	Very thin sides of the attractor	Heart rhythm almost periodic
Consistent increase/decrease in systolic and diastolic BP	No change in the attractor but change observed in the *u* variable	Overall increase/decrease in blood pressure

Application of machine learning methods to automate the identification of attractor features between ‘disease’ and ‘control’ groups, for example, will enhance the efficiency of extracting attractor feature ‘signatures’ that correlate with a particular cardiovascular phenotype. For example we have previously applied machine learning to identify the attractor differences between male and female ECG signals (Lyle *et al* 2018).

It now remains to be determined, through detailed investigations of annotated preclinical and clinical datasets, what each of the attractor features relates to physiologically. Our current knowledge of the physiological meaning of certain attractor features is summarised in table 1 and section 4. Furthermore, by generating a series of attractors for longer datasets we can see how the attractor signature might change with diurnal transitions, exercise, ageing, pharmacotherapy or disease.

Below we show real arterial waveform data from our experimental archive. These examples were sampled from rodents implanted with a radiotelemetry device sampling from the left carotid artery (Sand *et al* 2015) or healthy human volunteer data monitored with a finger tip blood pressure monitor (Finometer Midi, Finapres Medical Systems, Amsterdam, The Netherlands) (Silvani *et al* 2017). All experimental protocols had previously received full ethics approval and the original animal studies were conducted under a UK Home Office License and associated guidelines.

### 4.1. Attractor features and conventional pulse waveform measures

There are two features which can be extracted from the attractor reconstruction method which have exact correlates with conventional analysis, albeit they are calculated differently.

#### 4.1.1. Heart rate

Accurate heart rate detection is an essential part of the clinical management of patients and of biomedical research when investigating the impact of pharmacological or gene modifications. This has motivated collaborative efforts to enhance accurate beat detection, typically using ECG, particularly from noisy signals (Clifford *et al* 2016, Krasteva *et al* 2016).

Heart rate is conventionally extracted through automated identification of the QRS complex (ECG) or through peak or pulse onset detection (arterial blood pressure). In contrast to a peak detection method, a technique similar to autocorrelation (see Aston *et al* (2018)) is used to determine the average waveform cycle length measured in seconds. This uses the entire pulse waveform data rather than relying on feature detection of individual components. Average waveform cycle length is then used to generate the attractor and heart rate is calculated by dividing 60 seconds by the average waveform cycle length.

With noisy signals where there is high baseline wander, conventional analysis of heart rate through identification of, for example, R peaks may become compromised. However the attractor reconstruction method is not affected by changes in baseline wander, as described in section 3, figure 4 and appendix C.

**Figure 5.**
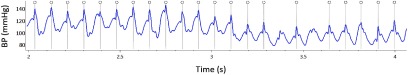
Mouse arterial blood pressure sampled at 1000 Hz with a radiotelemetry device. Illustrating the impact of baseline wander on automated peak detection.

We illustrate this difference in heart rate estimation in figure 5. The figure shows a two second window of mouse arterial blood pressure data and illustrates how baseline wander can affect automated peak detection. Manually counting the peaks in this window gives  ∼22.5 beats which equates to 675 bpm. Attractor reconstruction calculates the average cycle length to be 89 ms which equates to 674 bpm. However, as shown, automated peak detection can become compromised. In this example the two missed peaks result in a calculated heart rate of 600 bpm.

It remains to be tested whether the attractor reconstruction method of heart rate estimation is superior to other newly developed methods suitable for use with noisy physiological signals.

**Figure 6.**
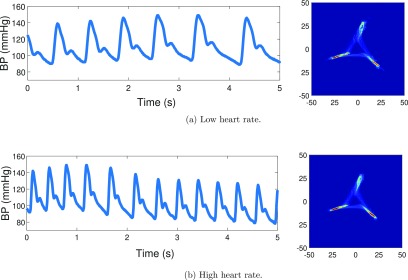
The shape and density of the attractor is not affected by heart rate. The arterial pulse waveform trace and corresponding attractor for traces with (a) resting heart rate and (b) with the heart rate artificially doubled (same trace). Human arterial pulse waveform data obtained by a non-invasive finger tip monitor (Finapres).

It is important to emphasize that the features of the attractor are not affected by heart rate. To illustrate, figure 6 shows the same pulse waveform trace from a healthy human volunteer but where the rate has been artificially increased in the lower panel. It can be seen that the resultant attractors are identical.

#### 4.1.2. Pulse pressure

Arterial pulse pressure is a function of left ventricular contractility, stroke volume and central arterial compliance. It can increase as a result of arterial stiffening arising from aging, it varies in response to fluid loading in ventilated patients and alters in syndromes such as sepsis (Esper and Pinsky 2014, Al-Khalisy *et al* 2015). As with heart rate, accurate estimation of pulse pressure is important in both the clinical and research setting.

**Figure 7.**
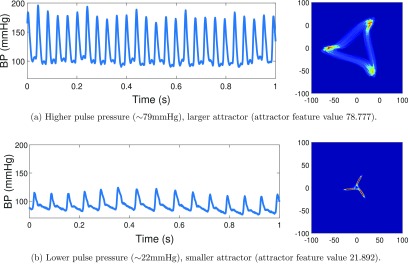
The overall size of the attractor directly relates to the pulse pressure of the arterial pulse waveform. As the pulse pressure increases, the corresponding attractor becomes larger. The absolute pressure values do not affect the attractor. The arterial pulse waveform trace and corresponding attractor for traces with (a) high pulse pressure during exercise and (b) low pulse pressure at rest. Human arterial pulse waveform data obtained pre and during exercise using a non-invasive finger tip monitor (Finapres).

The size of the attractor is directly proportional to the amplitude of the waveform (figure 7) and this can be determined again using the entire waveform signal by averaging the triangular attractor, finding the size of the resulting triangle, and scaling it appropriately (Aston *et al* 2018). The physiological meaning of the attractor’s rotation is described later. Also note, the differences in heart rate do not affect the attractor features.

Whether there is a difference in accuracy between conventionally derived or attractor reconstruction derived pulse pressure values remains to be tested.

### 4.2. Attractor features and pulse waveform morphology

We will now give three further examples of arterial pulse waveform morphology features and how they impact features of the attractor. These are not routinely quantified with conventional analysis.

#### 4.2.1. Waveform variability

It is well recognised that heart rate variability (HRV) has prognostic value (Camm *et al* 1996), yet despite decades of research, HRV as an analytical technique has not been implemented into routine clinical practice. This is partly because HRV analysis typically requires some form of data post processing and this would be impracticable in many clinical settings. In contrast, our method uses all of the data and does not require any processing (other than the removal of non-physiological artefacts). Interestingly, we have previously shown that the attractor reconstruction method can detect changes where HRV cannot (Aston *et al* 2014).

Attractor reconstruction does not give the same beat to beat measures as HRV but can give a measure of variability which tells the end user about the variability of the entire waveform. We have termed this feature ‘waveform periodicity’ which may provide more information about how the entire cardiac and peripheral vascular systems are behaving, e.g. during a transition from health to disease.

**Figure 8.**
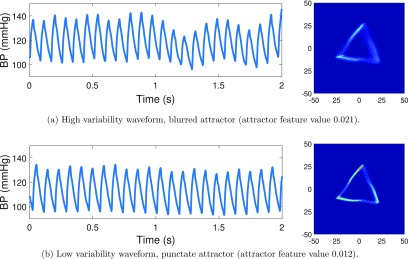
As an arterial pulse waveform becomes more periodic, the corresponding attractor becomes more defined. The arterial pulse waveform trace and corresponding attractor for traces with (a) high variability and (b) low variability. Mouse arterial pulse waveform measured with a radiotelemetry device.

In figure 8 we illustrate how waveform periodicity changes pre and post 1 mg kg^−1^ hydralazine in a single subject. Figure 8(a) shows a waveform of high variability and a corresponding diffuse attractor with blurred sides. In contrast figure 8(b) shows a waveform which has lower variability and this translates to a punctate attractor with well defined sides. Again, it is important to emphasize that the heart rate difference between the two traces does not impact on the attractor.

#### 4.2.2. Changes in waveform downstroke

**Figure 9.**
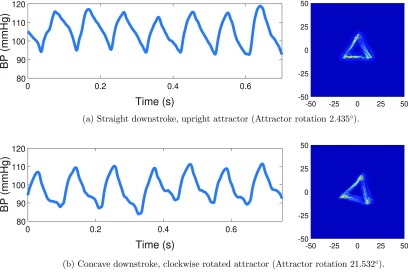
As the arterial pulse waveform downstroke becomes more concave (curved) so the attractor rotates clockwise. The pulse waveform trace and attractor for (a) a straight downstroke and (b) a concave downstroke. Mouse arterial pulse waveform measured with a radiotelemetry device both (a) before and (b) 30 minutes after a saline injection.

As resistance and compliance reduce, the downstroke of an arterial pulse wave can become more concave in shape (Alastruey *et al* 2014). Figure 9 illustrates this phenomenon from a single subject before and after a saline injection.

This data demonstrates that despite conventional measures of heart rate, systolic and diastolic pressure remaining comparable, the curvature of the downstroke in figure 9(b) translates to a clockwise rotation of the corresponding attractor. This is an example of where the attractor could enhance the sensitivity of detecting a change in response to a drug intervention or in the early stages of a disease when routine monitor readouts are similar.

#### 4.2.3. Variation in cardiac contraction

The upstroke of an arterial pulse waveform alters frequently as the nature of cardiac contraction varies with each beat. This can be quantified through measures such as *dP*/*dt* and is more commonly derived in a research rather than hospital setting. Direct intercardiac measures of pressure and contractility are also common in drug development.

**Figure 10.**
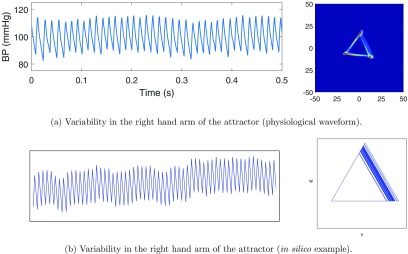
As the waveform upstroke gradient varies, the right hand arm of the attractor becomes wider. The waveform trace and attractor for (a) mouse arterial blood pressure and (b) an *in silico* waveform where upstroke gradient is altered whilst total period and downstroke is fixed. Mouse arterial pulse waveform measured with a radiotelemetry device. *In silico* trace and attractor adapted from Aston *et al* (2018).

Our investigations using the attractor reconstruction method on healthy human volunteer data revealed substantial movement on the right hand arm of the attractor. To identify the physiological correlate of this phenomenon, we created different shapes and features on *in silico* simulated waveforms and through systematic investigation identified that variability in the gradient of the upstroke of the waveform caused the greatest movement in the right hand side of the attractor (Aston *et al* 2018). An example of this can be seen in figure 10. Biologically this variability in the upstroke gradient likely correlates to beat to beat changes in the nature of cardiac contraction which can alter for a variety of physiological, pharmacological and/or pathological reasons.

There are of course many other waveform features and attractor correlates which we are systematically investigating.

## 5. Future validation and utility of attractor reconstruction

The primary aim of this paper was to provide an explanation of the attractor reconstruction method that could be more readily understood by those less experienced in signal processing but who regularly derive information from arterial pulse waves or other physiological waveforms. Whilst we have shown snapshots of how the method can be applied to such data, the attractor reconstruction approach necessarily requires full validation to identify the sensitivity and specificity of distinguishing between different clinical and/or experimental groups. As such, we are currently investigating the potential value of the method to provide more sensitive and earlier signals of cardiovascular change from animal models of disease along with using archived and prospective clinical datasets from both human volunteers and patients.

Ultimately, the method would need to function in real time to be clinically useful—but could be used retrospectively on research data. Further, as attractor signatures are identified for particular cardiovascular phenotypes, these would need to be coupled to readily understandable outputs for the end user (e.g. an alarm system) such that they could meaningfully facilitate clinical decision making. Whilst we have primarily focused on arterial pulse waveform data that would typically be obtained from indwelling catheters, it is important to remember that this method can be used on any physiological waveform, providing it is approximately periodic.

Future development of the attractor reconstruction method for clinical and research data includes determining optimal window lengths for different species and signal types alongside further refinement and exploration of attractor features used to identify cardiovascular phenotypes. To summarise, we have demonstrated that the attractor reconstruction method accomplishes the following.

1. Provides a new quantifiable representation of arterial waveform data in its entirety.
2. Uniquely quantifies changes in the shape and variability of the pulse waveform providing multiple readouts pertaining to specific waveform features.
3. Does not rely on the identification of specific features but uses the waveform in its entirety.
4. Does not provide a measure of absolute pressure (systolic, diastolic pressure).
5. Is unaffected by changes in physiological baseline wander.
6. Is heart rate independent.
7. Only requires removal of non physiological artifacts by end user.
8. May enhance the sensitivity of detecting cardiovascular changes that are not currently routinely measured.

Application of machine learning strategies would facilitate a more rapid identification of attractor features that distinguish between different groups and these features could be subsequently built into software as part of a detection device, essentially applying a pattern recognition approach which could be coupled to an alarm system.

However, we feel it is important to reverse translate the attractor features back to their physiological correlate where possible and this is achieved through the use of idealised simulated waveforms. Coupling the physiological root cause with the resultant attractor feature should enhance more rational interpretation of experimental data or clinical decision making.

## Figures and Tables

**Figure A1.**
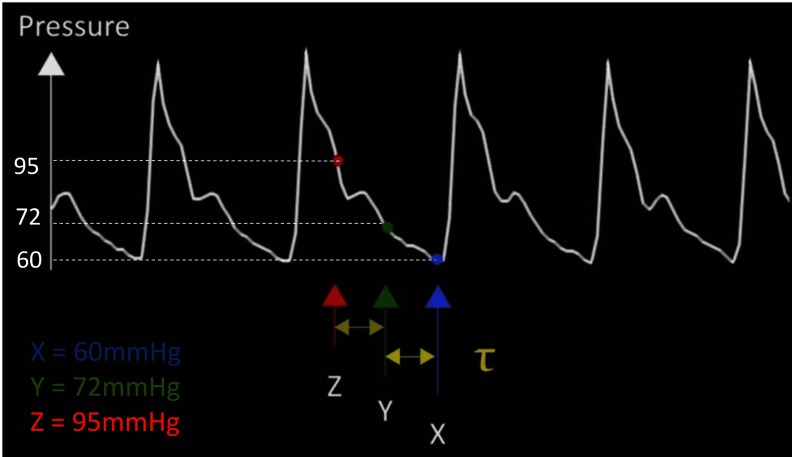
An arterial pulse waveform with three equally spaced points, *x*, *y* and *z*. The time delay (distance) between these points is equal and is denoted *τ*.

**Figure A2.**
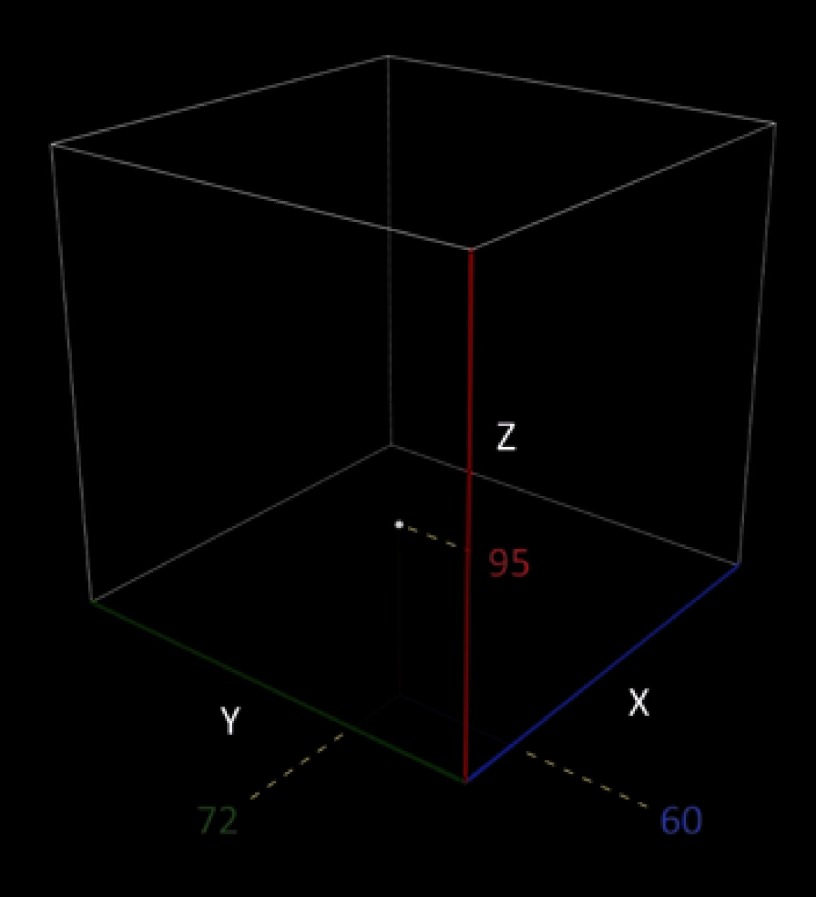
The three points *x*, *y* and *z* are used to create a single point in three-dimensional space.

**Figure A3.**
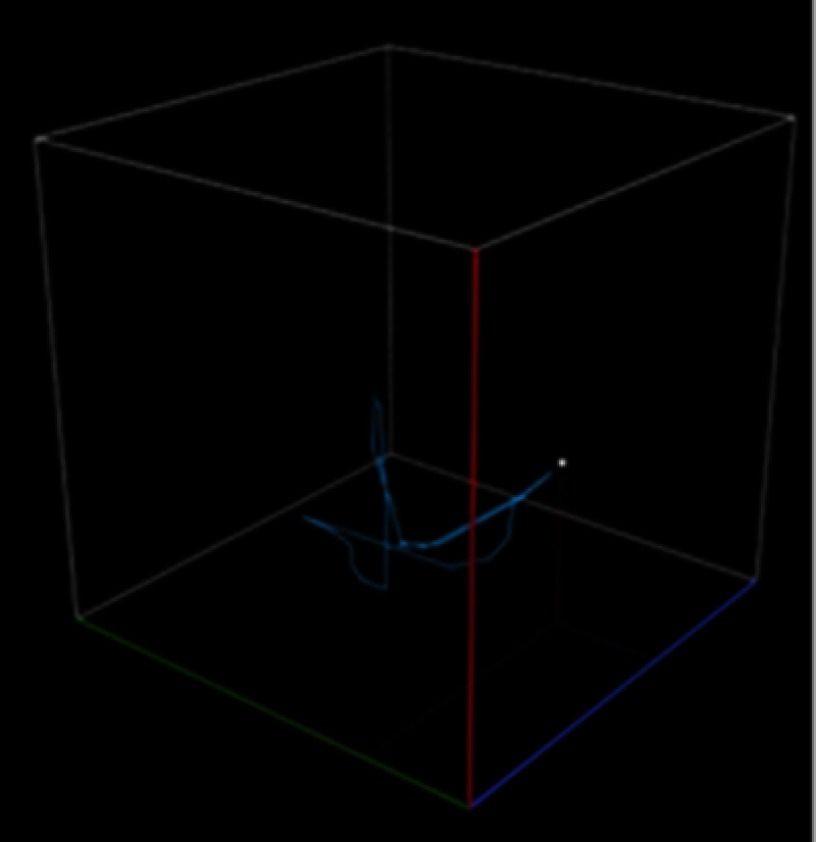
A single loop in a cube is generated when all three points have traversed one complete pulse wave.

**Figure A6.**
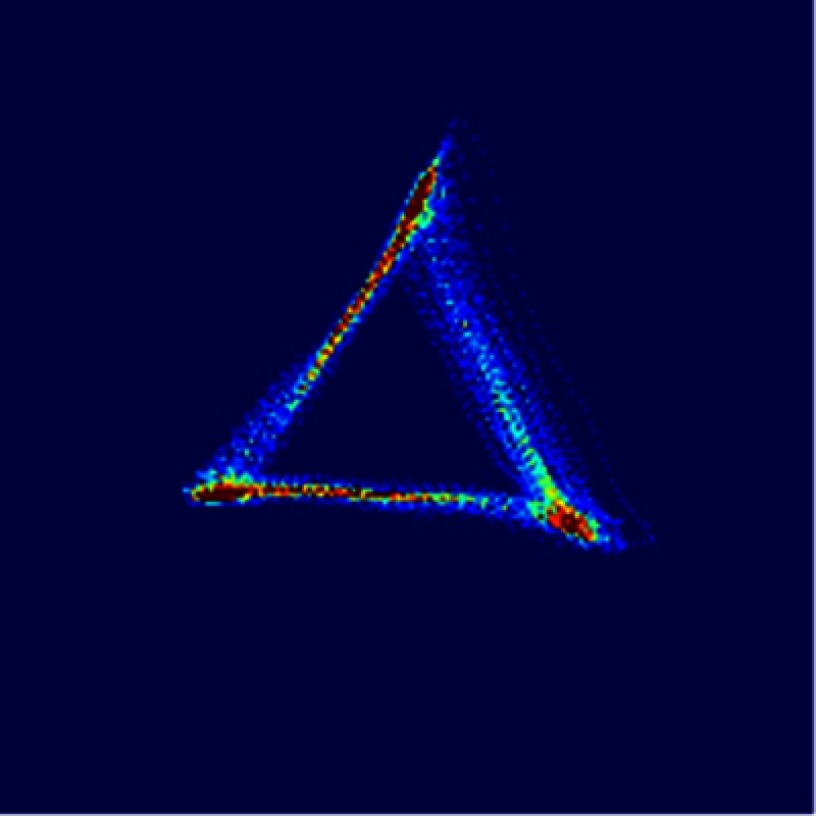
An attractor viewed in two-dimensions. Regions with high density (lots of overlap between loops of the attractor) are seen as red ‘hot spots’.

**Figure C1.**
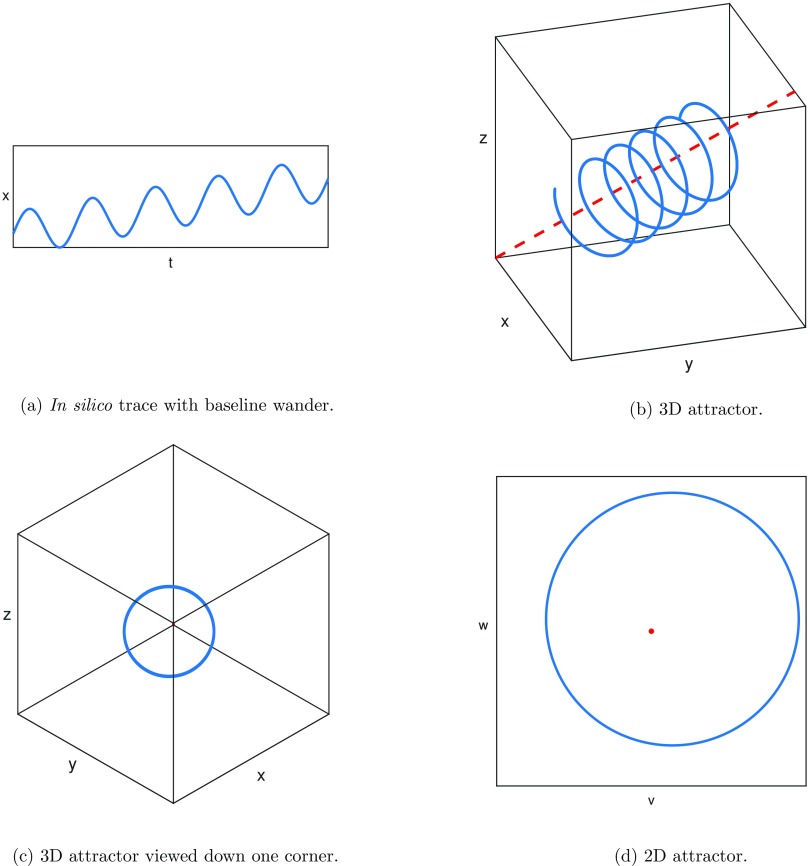
Explanation of why we view the attractor from one particular corner. Using the (a) initial waveform *x*, we obtain *y* and *z* and plot these to obtain the (b) three-dimensional attractor which has variation along the red dotted line. Viewing this from one corner (along the red dotted line) produces a (c) two-dimensional shape. To draw this new shape we define three new coordinates *u*, $v$ and *w* then plot $v$ and *w* to produce the (d) two-dimensional attractor. Variation along the red dotted line is no longer visible.

**Figure E1.**
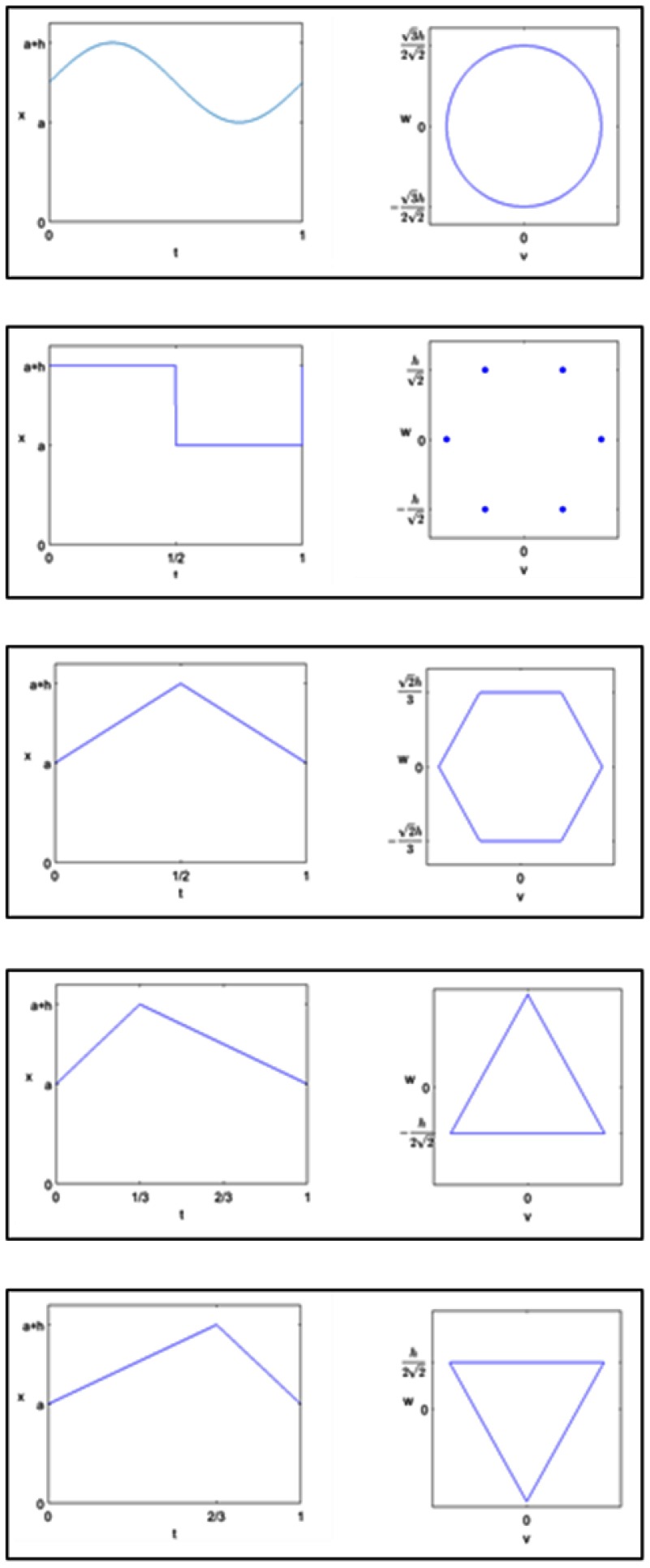
Sine waves and some simulated piecewise linear signals (left) with corresponding attractors (right).

## Appendix A. How is the attractor generated?

To explain this process to a non-mathematical audience, if we assume our arterial pressure wave is sampled at 125 Hz and the heart rate is 60 bpm, then it follows that we have 125 data points per beat or pulse wave. First, we randomly select a single data point on the waveform and denote this by *x*. We then define a second point at a fixed time delay *τ* behind *x* and denote this by *y*, and a final point two time delays ($2\tau$) behind *x* and denote this *z* (see figure A1). The time delay should be one third of the length of one pulse wave; thus if one beat is 1 s long, *τ* should be 333 ms.

This process extracts three numerical values (*x*, *y* and *z*) from the same pulse waveform allowing the data to be re-represented as a single point in three-dimensional phase space (figure A2). Next, by shifting each of the *x*, *y* and *z* coordinates forward to the next numerical value in the raw waveform data, this will slightly shift the point in the cube. The process can be repeated until all three points have each traversed all 125 numerical values of the single pulse wave, and are now positioned on the next pulse wave. This results in a loop, representing one pulse wave or ‘beat’, within three-dimensional phase space (figure A3).

By repeating this process for each and every pulse wave in a specified time window, numerous overlapping loops are generated—this is termed an ‘attractor’ (figure A4). Thus, the raw waveform data is re-represented in its entirety, but constrained within the three-dimensional space. However, quantification of the attractor features and relating those features to cardiovascular physiology would be challenging at this stage given its apparent chaotic structure.

This ‘chaos’ derives from the natural variability of a biological system. In other words, each pulse wave is slightly different to the next in terms of the baseline wander, the nature of the cardiac contraction, the resultant forward and backwards reflections and the resistance and compliance of the vasculature. In particular, the baseline wander seems to be the main source of the largest differences from beat to beat.

Had figure A4 been generated from an exactly periodic waveforms, for example a simulated waveform, it would have generated a single, identical overlapping closed loop with no variability or ‘noise’.

To address this noise issue for biological data, the next step rotates the three-dimensional cube, visualising the attractor diagonally from one particular corner (orange arrow figure A4). By looking straight down one corner of the cube, the three-dimensional cube is reduced to a two-dimensional image (figure A5).

This view generates a more defined structure and attractor shape. From this angle the effects of baseline wander in the original pulse wave are no longer visible (figure A5). Importantly, none of the original data has been deleted, we are just viewing it in a way that allows us to exclusively focus on the shape of the pulse waveform.

The final step is to add density using colour, such that for a given time window of data the degree of overlap between each consecutive attractor loop can be visualised and quantified (figure A6). A full explanatory video is available online^5^ 5 http://ehealth.kcl.ac.uk/cardiomorph/. .

## Appendix B. How can the attractor monitor changes over time?

Where attractor reconstruction is applied to data that spans a long period of time (e.g. hours or days), a suitable moving time window needs to be chosen. One attractor is generated for each time window such that a series of dynamically changing attractors is generated as the arterial pulse waveform shape changes over time.

If the moving time window is too short, each attractor may lack sufficient definition to enable meaningful measures to be derived. In contrast, if the window is too long, the density of the attractor may become saturated, such that subtle changes are missed.

We have previously selected a moving time window that incorporates around 100 pulse waves, this window is  ∼100 s for healthy human data (heart rate  ∼60–80 bpm) and  ∼10 s for mouse data (heart rate  ∼500–600 bpm). We found that this provides sufficient detail to allow quantification of changes over time, however detailed optimisation should always be conducted with different datasets.

## Appendix C. Why is the attractor viewed down one particular corner?

Viewing the attractor down one corner as illustrated in figure A4 results in the two-dimensional attractor that we take quantifiable measures from (figure A6). This direction of view is always the same.

Taking the simulated stream of waveform data *x* in figure C1(a) we calculate *y* and *z* coordinates using *τ* (this process is described in more detail in appendix A). Plotting *x*, *y* and *z* gives the three-dimensional attractor in figure C1(b). When we view the three-dimensional attractor from one corner it appears two dimensional and movement along the red dotted line is no longer visible (figure C1(c)).

To eliminate the movement along the red dotted line and obtain a two-dimensional attractor, we define three new coordinates *u*, $v$ and *w* (see Aston *et al* (2018)). Movement along the red dotted line is now represented as movement along the *u* axis. In these new coordinates, $v$ and *w* appear as the *new* vertical and horizontal axes respectively (figure C1(d)). However *u* is perpendicular to the page so is no longer visible. Therefore movement along the *u* axis will not be visible in this two-dimensional attractor. As shown in Aston *et al* (2018), figure 3, the movement along the *u* axis directly relates to baseline wander in a raw arterial pulse wave signal.

## Appendix D. How are the time delay values chosen?

Time delay *τ* is the distance between the original waveform points (*x*, *y* and *z*) chosen for attractor reconstruction (figure A1). The optimisation of the choice of time delay (*τ*) is described in detail elsewhere (Aston *et al* 2018).

The time delay itself is not fixed but rather self-adjusts as it tracks through each window of data (e.g. 100 s for healthy human data). This self-adjustment is based on the calculation of the average waveform cycle length (i.e. duration of each beat)—within that time window. If identical waveforms were generated mechanically, the time delay would remain fixed and the attractors would overlap exactly and maintain a defined structure and shape. In a biological setting, however, heart rate will inevitably vary and if *τ* did not adjust, the attractor would collapse and no longer be quantifiable.

Hence, the system adjusts *τ* accordingly: shortening with tachycardia and lengthening with bradycardia. This adjustment maintains a series of attractors with a defined ‘triangular’ shape (3 fold rotational symmetry) from which measurements can be taken.

## Appendix E. Why is the attractor triangular in shape?

The attractor reconstruction method applied to different waveforms can result in unintuitive attractor shapes, see figure E1. The triangular shape of the arterial waveform attractor is sometimes, incorrectly, assumed to relate directly to the almost triangular shape of an arterial pulse wave. The fact that an arterial pulse wave happens to be almost triangular in shape and the resultant attractor is also triangular in shape should be considered to be coincidental. For example, a change on the systolic upstroke of an arterial pulse wave (the left hand side of the waveform) will not impact the left hand arm of the attractor triangle. As described previously, the associations of pulse waveform morphology changes and the resultant feature changes in the attractor, can only be determined through systematic investigation. The mathematical proofs relating a piecewise linear signal to a simulated attractor have been previously described (Aston *et al* 2018).

## Glossary

Attractor features: Individual features of the attractor, e.g. the width of the arms, the angle of rotation.
Attractor reconstruction: Mathematical process of replotting waveform data in three-dimensional space using delay coordinates.
Attractor signatures: Combinations of attractor features that may correspond to a particular cardiovascular phenotype.
Periodic waveforms: Waveforms that repeat. Note biological data is unlikely to be 100% periodic as there is always some variability, it is therefore described as ‘approximately periodic’.
Sampling frequency (Hertz): The number of data points captured per second (100 Hz → 100 data points per second).
Simulated $\boldsymbol{in ~silico}$ signal: A waveform generated by a computer.
Three-dimensional phase space plotting: A method of plotting data points with three axes (*x*, *y*, *z*) in a cube for each time point as opposed to the more commonly used plot of (*x*, *y*) where *x* is time.
Time delay *τ*: Distance between points on the original waveform that are used for attractor reconstruction.
Time series data: Continuous data sampled over a given period of time.
Time window: Length of waveform used to generate one attractor. A long arterial pulse waveform recorded, for example, over several hours will have numerous overlapping time windows.
Waveform morphology: Features of the waveform’s shape.
Waveform variability: Difference between separate waveforms in a section of data.
